# Molecular Public Health for Infectious Disease Prevention and Control

**DOI:** 10.3390/microorganisms14102173

**Published:** 2026-09-26

**Authors:** Hirokazu Kimura, Ryusuke Kimura, Fuminori Mizukoshi, Aoi Shikichi, Hiroyuki Tsukagoshi, Haruyoshi Tomita, Akihide Ryo

**Affiliations:** 1 Advanced Medical Science Research Center, Gunma Paz University, Takasaki-shi 370-0006, Gunma, Japan; 2 Department of Health Science, Graduate School of Health Sciences, Gunma Paz University, Takasaki-shi 370-0006, Gunma, Japan; 3 Department of Infectious Disease Testing, Gunma Prefectural Institute of Public Health and Environmental Sciences, Maebashi-shi 371-0052, Gunma, Japan; 4 Department of Bioinformatics and Integrative Omics, National Institute of Infectious Diseases, Japan Institute for Health Security, Musashimurayama-shi 208-0011, Tokyo, Japan; 5 Department of Bacteriology, Graduate School of Medicine, Gunma University, Maebashi-shi 371-8511, Gunma, Japan; tomitaha@gunma-u.ac.jp

**Keywords:** molecular public health, public health microbiology, genomic surveillance, bioinformatics, genomic epidemiology, metagenomics, wastewater-based epidemiology, one health, antimicrobial resistance, artificial intelligence

## Abstract

Molecular technologies have transformed infectious disease surveillance, but their public health value depends on whether molecular signals can be converted into timely and effective action. Unlike molecular epidemiology, which primarily uses molecular variation to characterize distribution, relatedness, transmission, and evolution, molecular public health is defined here as an end-to-end operational framework that connects molecular evidence to population-level decisions, interventions, and evaluation. This narrative review integrates molecular detection, pathogen genomics, bioinformatics, metagenomics, wastewater and environmental surveillance, One Health monitoring, antimicrobial resistance genomics, multi-omics, and artificial intelligence. The framework progresses from detection and analytical interpretation to prediction, intervention, and feedback. Implementation maturity is heterogeneous: targeted PCR, short-read pathogen genomics, and bioinformatics-supported outbreak investigation are established in many defined use cases, whereas pathogen-agnostic metagenomics, multi-omics, and AI-enabled decision support remain more context dependent or emerging. Actionability is operationalized through analytical validity, epidemiological interpretability, sample-to-decision time relative to the intervention window, decision impact, and measurable post-intervention outcomes. Persistent barriers include sampling bias, uneven global capacity, incomplete metadata, insufficient standardization, governance concerns, and poorly defined trigger thresholds. The central criterion for the field should therefore be whether molecular information measurably improves prevention and control.

## 1. Introduction

Infectious disease microbiology developed around linking specific microorganisms to disease and around microscopy, staining, culture, biochemical identification, serology, and experimental criteria for microbial causation [1,2,3]. These approaches established the foundations of clinical microbiology and infectious disease epidemiology, but they were constrained by culture requirements, variable growth characteristics, limited discriminatory power, and difficulty identifying fastidious or phenotypically unidentifiable organisms [2,3,4]. Improvements in phenotypic identification, including matrix-assisted laser desorption ionization–time of flight mass spectrometry, shortened organism identification after culture, while multiplex assays broadened the number of pathogens detectable in a single clinical episode [5,6]. Collectively, microbiological diagnosis began to shift from descriptive observation of microbial phenotype toward rapid extraction of biologically specific information from clinical specimens [3,5,6].

The intellectual basis for the next transition was provided by twentieth-century molecular biology. The demonstration that deoxyribonucleic acid (DNA) carries heritable biological information and elucidation of its double-helical structure established a molecular framework for understanding microbial inheritance, mutation, and evolution [7,8]. Chain-termination sequencing made nucleotide sequence experimentally accessible, allowing microbial variation to be described at the level of individual bases rather than only by morphology, antigenicity, or biochemical behavior [9]. The polymerase chain reaction (PCR) transformed infectious disease diagnostics by enabling selective amplification of microbial nucleic acids directly from specimens and reducing dependence on cultivation [10,11]. Real-time PCR added rapid quantification and kinetic monitoring, making temporal changes in nucleic-acid concentration measurable parameters for diagnosis and surveillance [12]. These advances established a central premise of contemporary molecular microbiology: microbial identity, abundance, variation, and evolution can be inferred directly from molecular information [9,10,11,12].

High-throughput sequencing produced a further qualitative change because microbiological investigation was no longer limited to predefined targets or short genetic regions. Whole-genome sequencing can simultaneously provide information on pathogen identity, lineage, mutations, virulence-associated determinants, antimicrobial resistance, and genetic relatedness, thereby linking laboratory characterization directly to epidemiological investigation [13,14]. During the severe acute respiratory syndrome coronavirus 2 (SARS-CoV-2) pandemic, analytical systems such as Nextstrain, Pangolin, and UShER demonstrated that pathogen genomes could be classified, phylogenetically placed, and visualized at a scale compatible with near-real-time surveillance [15,16,17]. Large-scale genomic analyses further showed that sequence data could reconstruct repeated introductions, lineage replacement, and transmission dynamics at population level [18]. In healthcare settings, rapid sequencing integrated with epidemiological metadata has helped distinguish probable nosocomial transmission from unrelated introductions and has informed infection prevention and control decisions [19,20]. Thus, the unit of microbiological interpretation expanded from the individual organism and patient to transmission networks and pathogen populations [14,18,19,20].

This expansion made bioinformatics inseparable from modern public health microbiology because sequence generation alone does not produce epidemiological meaning. Raw molecular data must be transformed through quality control, read processing, mapping or assembly, consensus generation, variant calling, lineage assignment, phylogenetic reconstruction, functional annotation, and linkage with temporal, geographic, clinical, and exposure metadata [14,21,22]. These analytical steps determine whether a genomic difference represents technical noise, within-host variation, a new lineage, a transmission cluster, or a resistance-associated mutation [14,21,22]. Bioinformatics therefore functions not merely as computational support but as the interpretive engine that converts molecular measurements into structured evidence for epidemiological inference and public health action [14,21,22,23]. Reproducibility, version control, standardized reference datasets, and transparent uncertainty reporting become increasingly important as surveillance becomes automated, distributed, and high volume [21,22,24,25,26,27].

Moreover, the molecular field has simultaneously expanded beyond isolate-based genomics. Metagenomic next-generation sequencing can detect microbial nucleic acids without requiring a pathogen-specific hypothesis and therefore provides a route to identifying unexpected, difficult-to-culture, or previously unrecognized infectious agents [28,29,30,31]. It can also characterize viromes, bacteriomes, resistomes, and mixed microbial communities, although interpretation depends strongly on contamination control, database quality, taxonomic classification, and meaningful reporting thresholds [28,29,30,31]. Wastewater sequencing extends molecular observation from the patient to the community and can reveal transmission patterns or viral lineages incompletely represented by clinical testing [32,33,34]. Long-read sequencing further increases bacterial genomic resolution by resolving structural variation, plasmids, and mobile resistance elements that may be incompletely reconstructed by short-read sequencing [35]. Molecular surveillance can therefore observe pathogens across individuals, populations, microbial communities, and environmental reservoirs rather than within the boundaries of conventional clinical testing alone [28,29,30,31,32,33,34,35].

Thus, these technological changes have also altered the organization of public health microbiology. Contemporary systems require integration of laboratory science, bioinformatics, epidemiology, data engineering, governance, and communication. For public health practice, end-to-end turnaround from specimen collection to an interpretable report is an operationally critical performance measure, not sequencing speed in isolation [19,20,21,23]. The World Health Organization (WHO) Global Genomic Surveillance Strategy for Pathogens with Pandemic and Epidemic Potential 2022–2032 explicitly frames genomic surveillance as part of an end-to-end system that includes sampling, diagnostics, analysis, data sharing, and connectivity among disease-control programs [36]. WHO guidance for wastewater and environmental surveillance similarly emphasizes integration with other surveillance streams and implementation where results can support meaningful public health decisions [37]. These developments parallel the movement from public health genomics toward precision public health, in which increasingly granular biological and population data are used to improve the timing and targeting of prevention strategies [38,39].

Against this historical and technological background, an important conceptual gap remains. Molecular diagnostics, molecular epidemiology, genomic epidemiology, pathogen genomics for public health, and precision public health are established constructs, yet the literature often treats clinical genomics, wastewater and environmental surveillance, One Health, antimicrobial resistance, metagenomics, and computational prediction as partly separate domains [14,32,36,37,38,39,40]. We therefore define molecular public health as the integrated application of molecular microbiology, genomics, bioinformatics, epidemiology, and population surveillance to convert molecular information on pathogens and their ecological contexts into population-level risk assessment, decision-making, intervention, and evaluation. Molecular epidemiology is an essential component of this framework rather than a competing concept: it establishes molecular distributions and relationships, whereas molecular public health explicitly extends those inferences through an end-to-end operational pathway in which a molecular result becomes public health information only when it changes, or is capable of changing, what is done [14,19,20,36]. The framework also differs from precision public health in emphasis: precision public health uses increasingly granular biological and population data to improve the timing and targeting of prevention across health domains, whereas molecular public health centers specifically on molecular evidence concerning infectious agents, hosts, and reservoirs and its translation into infectious-disease prevention and control [38,39]. Accordingly, the principal evaluative criteria are actionability, timeliness, interpretability, reproducibility, equity, and measurable contribution to prevention and control rather than technological sophistication alone. The following sections therefore examine not only what each molecular technology can measure, but also how molecular evidence can be integrated into an operational decision cycle.

## 2. Review Scope and Literature Selection

This article was designed as a state-of-the-art narrative review rather than a formal systematic review. Literature selection was purposive and iterative, prioritizing publications from 2021 to 2026 while retaining foundational studies needed to explain the historical and analytical development of molecular public health. Targeted PubMed/MEDLINE searches and checks of authoritative public-health guidance were conducted for literature available through 14 September 2026, with particular attention to implementation, validation, equity, economic evaluation, wastewater normalization, and artificial intelligence. The aim was to achieve transparent, current coverage of the major conceptual and operational domains rather than exhaustive systematic enumeration. Search concepts were combined iteratively around molecular diagnostics, pathogen genomics, genomic surveillance, bioinformatics, genomic epidemiology, phylodynamics, metagenomics or pathogen-agnostic sequencing, wastewater or environmental surveillance, One Health, antimicrobial resistance genomics, host response or multi-omics, artificial intelligence or machine learning, implementation, public-health action, and infectious-disease control. Authoritative guidance from the World Health Organization and other public-health bodies was prioritized for questions of implementation, standardization, governance, and surveillance integration [36,37,41]. Priority was given to peer-reviewed primary studies, systematic or scoping reviews, implementation evaluations, and guidance documents that directly informed population surveillance, outbreak investigation, prevention, control, governance, or implementation. Studies focused solely on molecular mechanism or individual-patient diagnosis without a clear population or public-health implication were generally not prioritized. Because selection was purposive and iterative rather than prospectively systematic, no PRISMA record count or meta-analysis is presented; this limitation is stated explicitly so that readers can judge coverage and potential selection bias.

## 3. Conceptual Framework from Molecular Epidemiology to Molecular Public Health

For organizing the decision pathway, we use the established analytics terminology of descriptive, predictive, and prescriptive analysis, with an explicit analytical/interpretive stage inserted to reflect the requirements of infectious-disease surveillance [42]. This sequence is used as a heuristic for public-health translation rather than proposed as a new general analytics taxonomy. The descriptive stage identifies which pathogen, lineage, mutation, resistance determinant, or molecular signal is present; the analytical stage determines how those signals are related in time, place, host, and population; the predictive stage estimates what is likely to happen next; and the prescriptive stage links the resulting risk estimate to a feasible intervention. Molecular and genomic epidemiology contribute principally to the descriptive and analytical stages and increasingly to prediction. Molecular public health adds an explicit operational requirement that these stages be connected to a decision owner, intervention window, intervention, and post-intervention evaluation. It also broadens the surveillance architecture by integrating clinical, genomic, wastewater, food, animal, vector, and environmental signals where appropriate [32,33,34,36,37]. Table 1 summarizes the relationship between molecular public health and adjacent constructs. The progression from molecular detection to population-level public health action is summarized in Figure 1.

## 4. Bioinformatics and Data Integration: The Interpretive Engine of Molecular Public Health

Because the molecular public health framework depends on interpretation rather than sequence generation alone, bioinformatics occupies a central position between the laboratory and the public health decision [14,21,22,23]. Routine genomic workflows typically include raw-read quality assessment, adapter and low-quality trimming, contamination screening, reference mapping or de novo assembly, consensus generation, variant calling, lineage or sequence-type assignment, phylogenetic placement, and functional annotation [14,22,44]. Reproducible workflow managers and analysis environments such as Snakemake, Nextflow, and Galaxy allow complex pipelines to be versioned, automated, and transferred between laboratories, thereby reducing analytical heterogeneity [24,25,26]. For public health use, these computational outputs must be integrated with curated metadata and rendered as interpretable products such as cluster reports, lineage-frequency summaries, resistance alerts, phylogenetic visualizations, or dashboards [15,16,17,21,23]. Accreditation, validated pipelines, external quality assessment, version-controlled reference datasets, and explicit reporting of uncertainty are therefore essential components of implementation rather than optional technical refinements [27,45]. Recent implementation frameworks increasingly treat end-to-end genomic surveillance—including informatics and interpretation—as a public health service that should be evaluated for timeliness, reproducibility, and impact on control decisions [43,45,46]. The end-to-end pathway from sampling and laboratory outputs through bioinformatic interpretation to public health action is illustrated in Figure 2.

The principal bioinformatic functions required to convert molecular data into interpretable public health intelligence are summarized in Table 2.

## 5. Molecular Detection and Quantification Technologies

Reverse transcription quantitative PCR (RT-qPCR) is widely used for predefined-target molecular surveillance because it is rapid, sensitive, scalable, and well standardized [3,12,36,37]. Digital PCR provides absolute quantification without reliance on a standard curve and can improve precision for low-copy targets or inhibitor-rich matrices [51]. Multiplex molecular panels increase syndromic breadth but remain constrained by assay design [6], whereas CRISPR-based detection offers rapid, potentially field-deployable testing, although sample processing, multiplexing, quantitative workflows, and end-to-end standardization remain implementation challenges [52,53,54]. These approaches do not have equivalent implementation maturity. For the purposes of this review, established denotes routine public-health use in multiple defined settings; context-dependent denotes operational use with substantial pathogen-, infrastructure-, or jurisdiction-specific constraints; and emerging denotes applications for which routine population-level implementation or demonstrated decision impact remains limited. These descriptors refer to implementation maturity rather than analytical validity and should not be interpreted as a universal ranking. The principal molecular technologies used across the molecular public health continuum are summarized in Table 3.

Indicative implementation maturity: established for targeted PCR/multiplex PCR and short-read WGS in defined surveillance or outbreak applications; established but context-dependent for dPCR and amplicon sequencing; context-dependent and expanding for long-read/hybrid sequencing; and generally emerging or highly context-dependent for pathogen-agnostic metagenomics and CRISPR-based integrated surveillance [14,43,53,54,58,60].

## 6. Next-Generation Sequencing and Pathogen Genomic Surveillance

Whole-genome sequencing provides high-resolution information on pathogen variation and has become a core platform for lineage surveillance, outbreak investigation, source attribution, and resistance characterization [13,14]. Short-read sequencing remains advantageous for high accuracy and throughput, whereas long-read sequencing can resolve structural variation, complete genomes, plasmids, and other mobile elements that are difficult to reconstruct from short reads alone [35]. Portable sequencing demonstrated during Ebola and subsequent outbreaks that genomic surveillance can be moved closer to the field and can provide information during an ongoing public health emergency [56]. National and regional programs now increasingly emphasize standardized sampling, automated bioinformatics, data integration, and rapid interpretation rather than sequencing capacity in isolation [27,36,43,45,46].

## 7. Genomic Epidemiology and Phylodynamics

Genomic epidemiology integrates pathogen sequence data with temporal, geographic, and host information to infer introductions, clusters, transmission patterns, and pathogen movement [14,18]. SARS-CoV-2 studies demonstrated that dense genomic sampling can reconstruct epidemic establishment and lineage dynamics at national scale, while growth-rate and phylodynamic analyses can identify variants with altered transmission characteristics [18,47,48,49]. Similar approaches have been used to characterize the international emergence and microevolution of mpox virus and other rapidly spreading pathogens [50]. Nevertheless, genomic similarity is not proof of direct transmission, and public health interpretation must explicitly account for incomplete sampling, evolutionary rate, epidemiological plausibility, and uncertainty [14,19,20].

## 8. Metagenomics and Pathogen-Agnostic Surveillance

Metagenomic sequencing extends molecular surveillance beyond predefined targets and is therefore particularly relevant to difficult-to-diagnose infections and preparedness for emerging or re-emerging pathogens [28,29,30,31,57,58]. Public health pathogen-agnostic sequencing programs have highlighted both its potential and its operational constraints, including host-background nucleic acid, contamination, sequencing depth, reference-database bias, computational demand, and difficulties assigning clinical or epidemiological significance to low-abundance findings [30,57,58]. Environmental applications are also expanding, with metagenomic sequencing detecting respiratory and enteric viral signals in air collected from congregate settings [59]. The central implementation requirement is a validated interpretive pathway that distinguishes unusual but genuine signals from analytical noise and defines when a finding should be escalated for confirmatory testing or epidemiological investigation [30,31,57,58].

## 9. Wastewater and Environmental Surveillance

Wastewater and environmental surveillance (WES) provides population-level molecular information independent of individual healthcare-seeking behavior and therefore complements case-based surveillance [32,33,34,37,61]. During the coronavirus disease 2019 (COVID-19) pandemic, wastewater sequencing detected cryptic SARS-CoV-2 lineages and provided information on variant circulation that was not fully captured by clinical sequencing [32,33]. The approach is now being generalized to other respiratory and enteric pathogens and to antimicrobial resistance, but quantitative comparison is affected by shedding biology, catchment structure, dilution and inflow, recovery efficiency, sampling design, and the denominator used to represent the contributing population [34,37,61,62]. Normalization strategies include flow- or mass-load normalization, fecal-strength or fecal-indicator markers, process/recovery controls, and estimated catchment population; these choices can materially alter between-site comparisons, and no single normalization method is universally optimal [61,62]. Real-world reviews indicate that WES can trigger testing, communication, outbreak investigation, or other interventions, but detection-to-action pathways remain heterogeneous and are often not prospectively defined [63]. Future systems therefore require explicit action thresholds, transparent reporting of normalization assumptions and uncertainty, and integration of wastewater signals with clinical, genomic, and epidemiological surveillance [37,61,62,63].

## 10. One Health Molecular Surveillance

The ability to compare molecular information across human, animal, food, and environmental sources makes One Health a natural extension of molecular public health [36,40]. Genomic surveillance can help identify zoonotic spillover, distinguish sustained human transmission from repeated animal-to-human introductions, and connect foodborne cases with agricultural or environmental sources [14,40,64]. Whole-genome sequencing has already transformed foodborne disease surveillance by improving discrimination among closely related isolates and strengthening outbreak detection and source attribution [64]. The major remaining challenge is interoperability because different sectors frequently use incompatible metadata standards, sampling strategies, legal frameworks, and reporting systems [36,40]. The integration of molecular surveillance across human, animal, food, environmental, and vector domains is illustrated in Figure 3.

**Figure 3:**
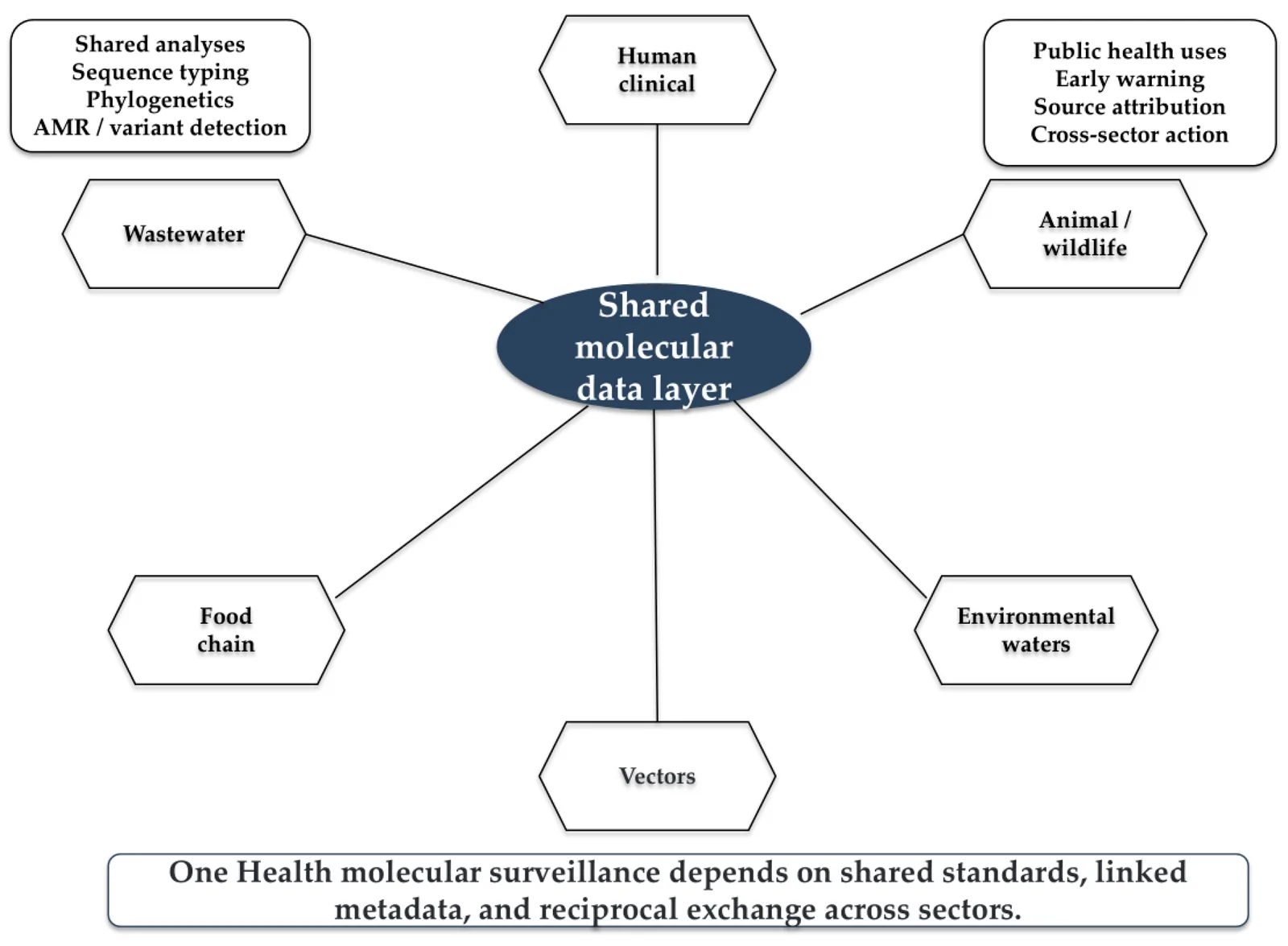
One Health molecular surveillance across human, animal, food, environmental, wastewater, and vector domains. Shared molecular methods and interoperable metadata support early warning, source attribution, and coordinated cross-sector action. The major surveillance domains and their potential public health applications are summarized in Table 4.

**Table 4:** One Health Sampling Domains and Their Roles in Molecular Public Health.

Surveillance Domain	Typical Specimens or Sources	Main Molecular Objectives	Representative Public Health Applications	Key References
Human clinical surveillance	Diagnostic specimens, isolates, syndromic panels	Detect pathogens, track variants or antimicrobial resistance (AMR), investigate transmission	Case confirmation, cluster investigation, hospital infection prevention and control (IPC), national surveillance	[14,19,20,43,46]
Wastewater surveillance	Sewage influent, sewer catchments, sludge	Monitor community-level pathogen circulation and temporal trends	Early warning, trend monitoring, complementary community surveillance	[32,33,34,37,61,63]
Food and food-chain surveillance	Foods, food-processing environments, production chains	Source attribution and genomic linkage between sources and human cases	Foodborne outbreak investigation, traceback, and source control	[40,64]
Animal and wildlife surveillance	Livestock, companion animals, wildlife reservoirs	Detect zoonotic spillover signals and compare strains across sectors	One Health risk assessment and cross-species outbreak investigation	[36,40]
Vector surveillance	Mosquitoes, ticks, other arthropod vectors	Detect vector-borne pathogens and characterize circulating lineages	Targeted vector control and ecological risk monitoring	[36,40]
Environmental surveillance	Surface waters, built environments, healthcare environments	Detect contamination, silent circulation, or environmental persistence	Environmental source investigation and intervention evaluation	[37,59,61,63]

## 11. Molecular Public Health Approaches to Antimicrobial Resistance

Antimicrobial resistance (AMR) is particularly suited to a molecular public health framework because resistance can spread through clonal expansion, mutation, horizontal gene transfer, mobile genetic elements, and movement across healthcare, community, food, animal, and environmental reservoirs [35,40,65]. Genomic analysis can identify resistance determinants and high-risk clones, while long-read sequencing can resolve plasmid context and help distinguish clonal transmission from dissemination of mobile resistance elements [35,40]. However, genotype and phenotype are not interchangeable. The presence of a resistance determinant may not produce the expected phenotypic resistance because expression, regulatory effects, gene context, epistasis, breakpoint definitions, and incomplete reference databases can alter the observed phenotype; conversely, resistance may arise through mechanisms not represented in current reference sets [66]. Machine-learning methods increasingly use genome content and sequence variation to predict antimicrobial susceptibility or resistance, but performance depends on representative phenotype-linked training data, organism- and drug-specific validation, and external evaluation [66]. Genomic prediction should therefore complement rather than automatically replace phenotypic antimicrobial-susceptibility testing when individual treatment decisions are involved. Public health value is achieved when validated molecular findings influence stewardship, empirical-treatment policy, outbreak control, environmental investigation, or prioritization of emerging resistance threats [40,65,66].

## 12. Beyond Genomics: Multi-Omics and Host-Response Surveillance

Pathogen genomes do not capture the full biology of infection, and transcriptomic, proteomic, metabolomic, and serological approaches can provide complementary information on host response, severity, immune status, and disease heterogeneity [67,68]. COVID-19 multi-omic studies, for example, identified coordinated immunologic and metabolic states associated with disease severity and transitions between clinical states [67,68]. These findings illustrate biological and risk-stratification potential, but most remain discovery- or research-stage observations rather than validated population-level action triggers [67,68]. For public-health implementation, high-dimensional signatures must be reduced to robust assays or reproducible computational outputs, prospectively validated in the intended population, generated within a relevant intervention window, and linked to a defined decision. Multi-omics should therefore be regarded as an emerging augmentation to pathogen-centered molecular surveillance rather than an operational equivalent of routine PCR or established genomic surveillance. Its most plausible near-term role is targeted host-risk stratification or mechanistic enrichment of surveillance in settings where the added information changes prioritization, resource allocation, or evaluation of interventions.

## 13. Artificial Intelligence and Computational Public Health

Artificial intelligence (AI) and machine learning can support selected molecular public-health tasks, including AMR prediction, anomaly detection, forecasting, and integration of heterogeneous surveillance streams [66,69,70]. A concrete molecular-data application is genome-based AMR prediction, in which machine-learning models infer susceptibility from genome content or sequence variation; performance is strongly dependent on organism, antimicrobial, training-data composition, and external validation [66]. At the broader surveillance level, recent reviews describe AI-enabled early-warning systems that integrate epidemiological, environmental, web, wastewater, and sometimes genomic data, while also showing that much of the published evidence remains retrospective, proof-of-concept, or locally validated rather than prospectively embedded in routine public-health decision pathways [69,70]. This implementation gap is itself important. A high-performing model should not be considered actionable until it has been externally validated in independent populations, prospectively evaluated in the intended workflow, calibrated for the decision threshold, assessed for bias and interpretability, and monitored for data or concept drift [69,70]. Operational evaluation should test whether the model improves time to detection, prioritization, resource allocation, or intervention outcomes compared with existing surveillance. AI is therefore best regarded as an analytical accelerator within the molecular public-health architecture, with accountability remaining with the public-health system using the model.

## 14. Translating Molecular Data into Infectious Disease Control

The defining feature of molecular public health is translation from molecular signal to public health action [14,36]. Genomic and other molecular data can support outbreak detection, transmission-chain investigation, targeted screening, cohorting, ward-level infection prevention and control (IPC), vaccination strategies, food traceback, source remediation, and antimicrobial stewardship when the analytical result is linked to an operational decision [19,20,36,40,63,65]. We define actionability as the condition in which molecular information has sufficient analytical validity, epidemiological interpretability, timeliness, and linkage to a feasible intervention to change a public-health decision within the relevant action window. This definition deliberately does not impose a universal numerical cutoff: an acceptable turnaround time, genomic distance, wastewater increase, or predictive probability depends on pathogen biology, sampling density, surveillance purpose, intervention availability, and jurisdiction. Action thresholds should therefore be prospectively specified and locally validated rather than retrospectively inferred after an outbreak [36,37,63].

### Operationalizing Actionability

Actionability can nevertheless be measured through a common set of dimensions. Candidate metrics include sample-to-decision time relative to the intervention window; the proportion of molecular reports that confirm, modify, escalate, or de-escalate a public-health action; concordance with epidemiological or phenotypic evidence; discrimination and calibration when predictive models are used; incremental yield over conventional surveillance; and post-intervention changes in transmission, shedding, cases, resistance, or response time [19,20,36,43,45,63]. These metrics should be selected before implementation and interpreted against a pre-specified baseline or counterfactual whenever feasible. Table 5 provides a practical measurement framework.

## 15. Challenges: Standardization, Ethics, Equity, and Governance

Technical barriers include sampling bias, insufficient denominators, incomplete metadata, contamination, pipeline heterogeneity, slow turnaround, and inconsistent reporting standards [27,36,43,45,46]. Implementation also depends on total resource requirements rather than sequencing reagent cost alone: platform acquisition and maintenance, batching and throughput, quality systems, data storage, bioinformatics, workforce, confirmatory testing, and reporting infrastructure all contribute to sustainability [45,60,71]. Comparative economic evidence remains incomplete. A 2023 systematic review found that the economic evidence supporting broad front-line WGS implementation was limited and heterogeneous, reinforcing the need to evaluate costs together with decision impact rather than assuming that greater molecular resolution is automatically cost-effective [71]. Metagenomic surveillance can provide pathogen-agnostic breadth, but its computational and financial burden is particularly relevant in resource-scarce settings, where targeted molecular assays or regionalized sequencing may answer the public-health question more efficiently [72].

Technical success also does not guarantee public-health impact. In the multicentre COG-UK HOCI prospective study, rapid SARS-CoV-2 sequencing changed IPC practice in a subset of hospital-onset cases but did not produce a statistically significant reduction in nosocomial infection incidence overall; the practical effect was greater when reports were returned within five days, whereas high case volume and constrained response capacity limited implementation [20]. Similarly, wastewater studies show that pathogen detection can lead to testing, communication, outbreak investigation, or other actions, but the link between detection and intervention remains inconsistent across settings [63]. These mixed outcomes illustrate why turnaround, decision ownership, response capacity, and outcome evaluation must be treated as part of the molecular system rather than as external implementation details.

Equity requires a resource-adaptive rather than technology-maximal approach. The preferred method should be the least complex platform that can answer the defined public-health question reliably and within the action window. Practical adaptations include sentinel rather than exhaustive sampling, targeted PCR or amplicon approaches when adequate, regional sequencing hubs, shared bioinformatics, transferable validated workflows, minimum interoperable metadata sets, workforce development, and integration with existing surveillance programs [36,72,73,74]. Unequal sequencing intensity also creates inferential bias: regions with limited capacity contribute fewer genomes and metadata, potentially distorting apparent lineage distributions, transmission routes, and AMR patterns [73,74,75]. Equity and representativeness should therefore be treated as measurable properties of surveillance performance, not only as downstream ethical considerations.

The major implementation challenges and corresponding practical responses are summarized in Table 6.

## 16. Future Perspectives Toward Closed-Loop Predictive Molecular Public Health

The next stage of molecular public health will move beyond retrospective characterization toward closed-loop systems in which surveillance generates molecular signals, bioinformatics converts those signals into structured risk information, predictive models estimate likely trajectories, decision-makers intervene, and subsequent surveillance measures the effect of intervention [14,36,39]. The SARS-CoV-2 experience demonstrated both the feasibility of global genomic surveillance and the consequences of unequal sequencing intensity, incomplete metadata, and variable integration with decision-making [18,75]. Future systems may combine pathogen-agnostic surveillance, real-time sequencing, wastewater and environmental monitoring, long-read genomics, host-response data, and multimodal AI within interoperable architectures, but such components should be adopted when they add validated decision value rather than simply increase technological complexity [31,35,37,58,69,70]. Progress should therefore be measured by earlier detection, more reliable interpretation, faster or better-targeted decisions, equitable coverage, and demonstrable intervention impact. As an illustrative operational workflow, consider a hospital in which two temporally linked hospital-onset infections trigger rapid sequencing under a pre-specified IPC protocol. Sequence and metadata integration first determines whether the cases are compatible with a shared transmission cluster; the report is then returned within the locally defined IPC action window and linked to a decision rule for targeted screening, cohorting, environmental investigation, or reinforcement of precautions. Subsequent molecular and epidemiological surveillance measures new cluster-associated cases over a defined follow-up period. If transmission does not decline, the decision rule or intervention is revised. The relevant performance measures are therefore not only sequence quality or phylogenetic resolution but also time to report, proportion of reports changing IPC, cluster-associated incidence, and the post-intervention trajectory [19,20,43,45]. The exact time and genomic thresholds are pathogen- and setting-specific; the closed-loop principle is the prospective linkage of signal, decision, intervention, and feedback. This closed-loop decision architecture is illustrated in Figure 4.

**Figure 4:**
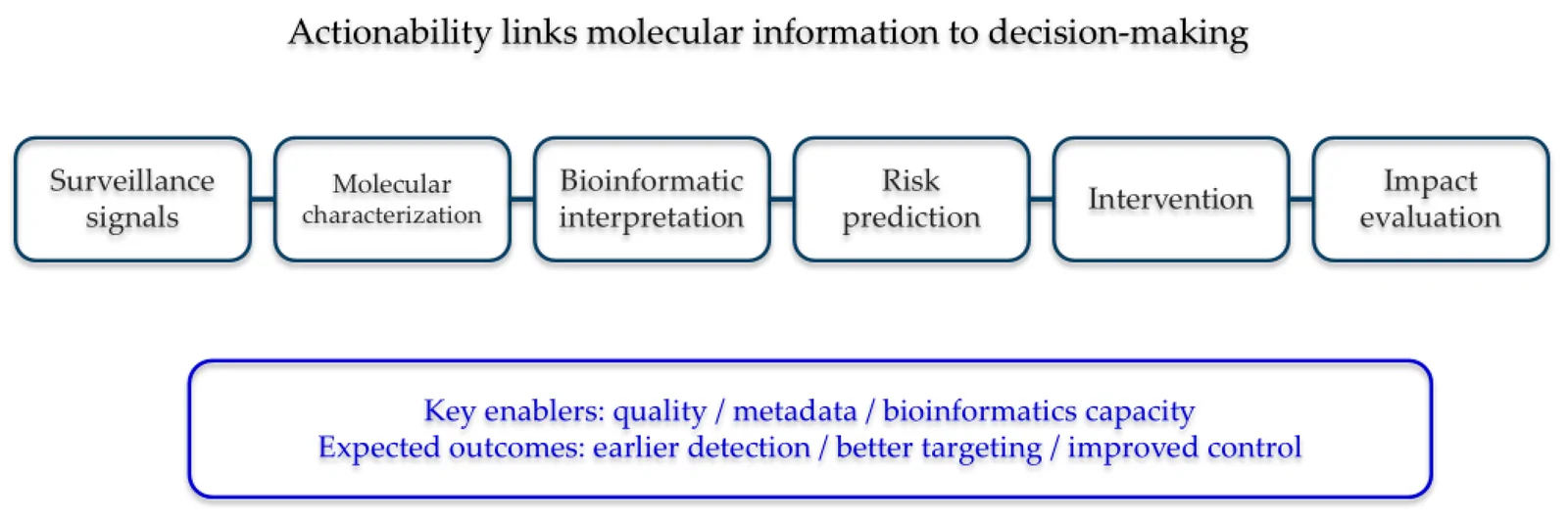
Closed-loop framework for predictive molecular public health. Surveillance, interpretation, prediction, intervention, and impact evaluation are linked by actionability and iterative feedback. The major stages, enabling factors, and expected outcomes of this closed-loop framework are summarized in Table 7.

**Table 7:** Conceptual Progression from Molecular Observation to Public Health Action.

Public Health Function	Typical Molecular Input	Decision or Action Supported	Example of Outcome to Evaluate	Key References
Detection	Targeted assays, sequencing, or metagenomics	Confirm that a pathogen or molecular signal is present	Time to recognition of a new event or outbreak	[3,14,36,55]
Characterization	Genomic lineages, mutations, antimicrobial resistance (AMR) determinants	Determine what is circulating and whether it differs from previous strains	Resolution of variant tracking or resistance monitoring	[13,14,36,43]
Interpretation	Phylogenetics, cluster analysis, metadata linkage	Judge whether cases are related or whether signals suggest broader spread	Accuracy of cluster identification or source attribution	[14,15,16,17,18,19,20,21,22,43]
Prediction	Temporal models, genomic trends, wastewater trajectories	Estimate what may happen next or where risk is increasing	Improved early warning or prioritization of response	[39,43,49,69,70]
Intervention	Integrated dashboards and action thresholds	Trigger infection prevention and control (IPC), targeted testing, vaccination, communication, or policy adjustment	Speed and appropriateness of implemented response	[19,20,36,37,43,63]
Evaluation	Post-intervention molecular and epidemiological data	Assess whether intervention altered transmission, circulation, or risk	Measured reduction in shedding, cases, spread, or response time	[20,43,45,63]

## 17. Conclusions

Molecular public health is best understood as an operational architecture for converting molecular evidence into population-level prevention and control, not as a replacement for molecular or genomic epidemiology. Across the evidence reviewed, targeted molecular detection, short-read pathogen genomics, standardized bioinformatics, and genomics-supported outbreak investigation already have established roles in defined public-health settings, whereas pathogen-agnostic metagenomics, multi-omics, and AI-enabled decision support have more heterogeneous implementation maturity. Wastewater, long-read, and One Health approaches occupy context-dependent positions: their technical value is increasingly demonstrated, but action thresholds, standardization, infrastructure requirements, and integration with conventional surveillance remain variable [35,36,37,43,60,63]. The principal limitations are therefore not only analytical. Sampling bias, turnaround, metadata quality, interoperability, workforce and infrastructure, governance, cost, and unequal global representation determine whether a molecular signal can actually change a decision [20,45,71,72,73,74]. Key knowledge gaps include prospectively validated action thresholds, comparative implementation and economic evaluations, external and prospective validation of predictive models, and evidence that molecularly informed interventions improve outcomes. The practical implication is that future progress should be judged by validated decision impact, timeliness, equity, and measurable improvement in prevention and control rather than by the volume or technological sophistication of molecular data generated.

## Figures and Tables

**Figure 1:**
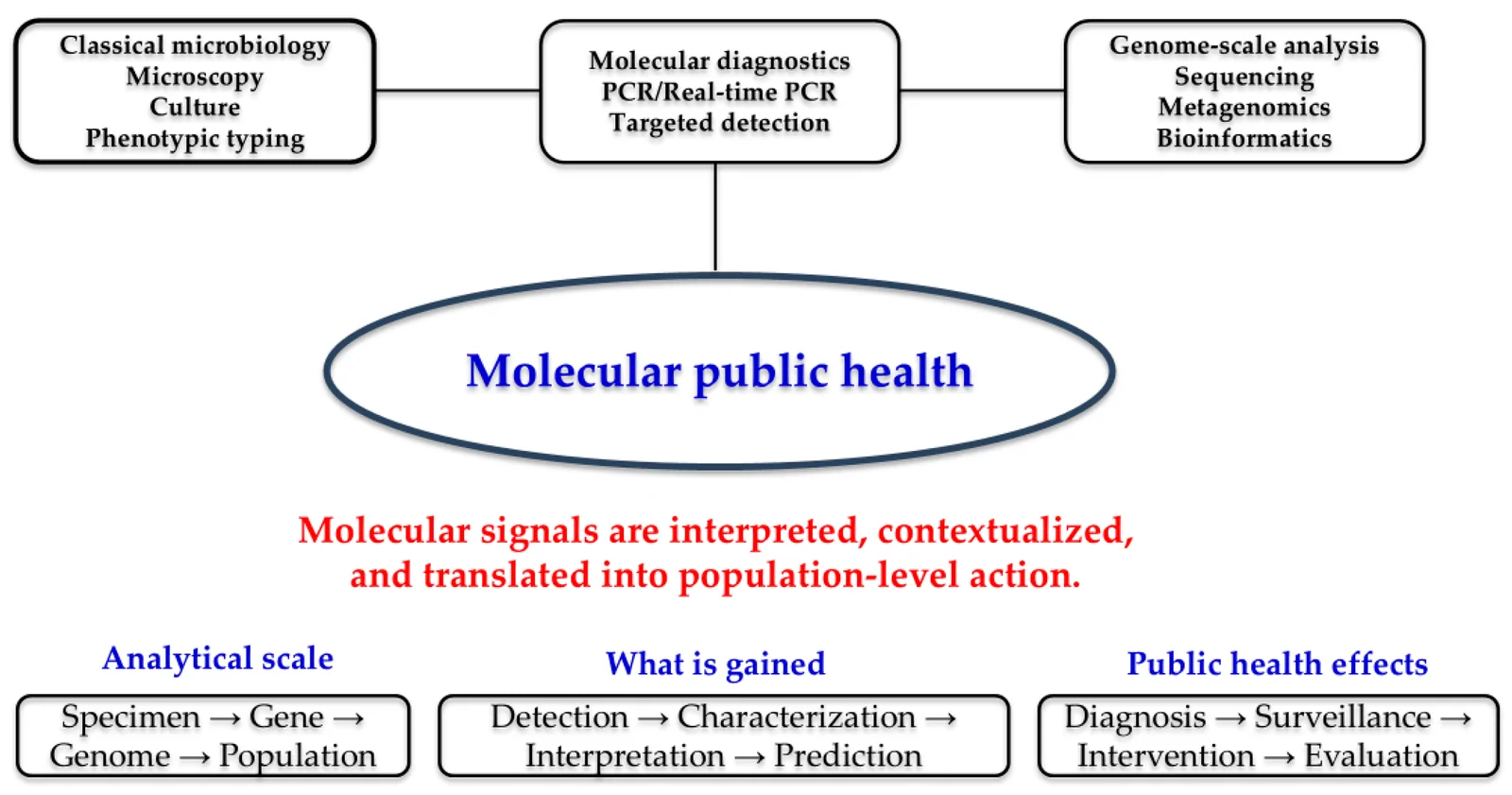
Conceptual progression from classical microbiology to molecular public health. The framework shows the expansion from specimen- and gene-level detection toward genome- and population-level interpretation, prediction, intervention, and evaluation.

**Figure 2:**
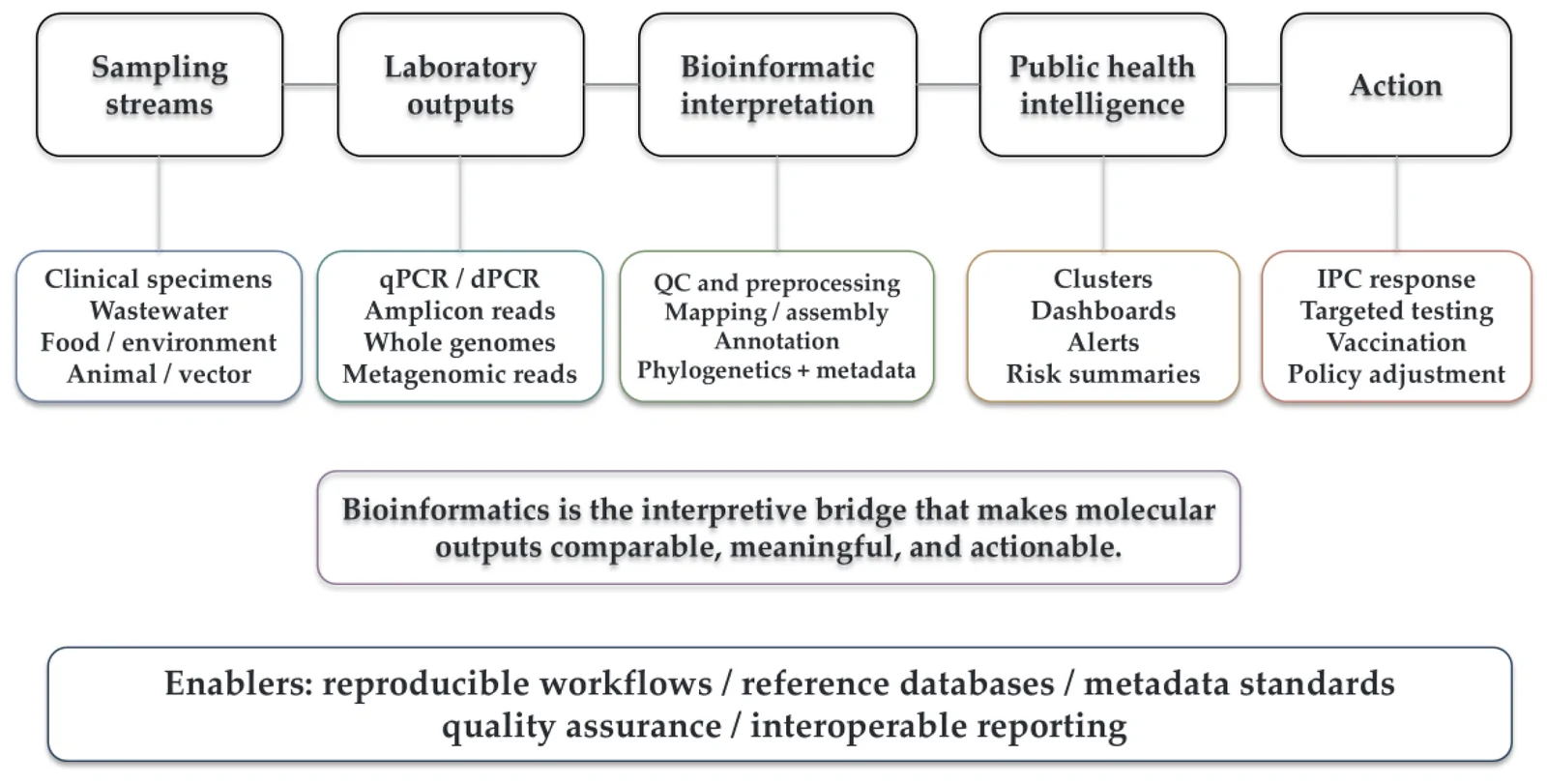
End-to-end workflow from molecular sampling to public health action. Molecular outputs from clinical, environmental, food, and animal/vector sources are transformed by bioinformatics and metadata integration into decision-ready public health intelligence.

**Table 1:** Comparison of Molecular Public Health with Adjacent Constructs.

Relationship to/Added Value of Molecular Public Health	Usual Operational Endpoint	Core Focus	Construct
Input layer only; population interpretation, intervention, and outcome evaluation are not inherent [3,10,11,12].	Patient/specimen diagnostic result; may feed aggregate surveillance	Detection or quantification of a defined molecular target	Molecular diagnostics
Core descriptive/analytical component; molecular public health explicitly links inference to decisions, intervention, and evaluation [14,18].	Inference on transmission, relatedness, and evolution	Distribution and relatedness of molecular variants across person, place, and time	Molecular epidemiology
Major genomic component; molecular public health additionally integrates non-genomic molecular streams and a closed intervention-feedback loop [14,19,20,43].	Outbreak/cluster inference and genomic surveillance	Whole-genome lineages, clusters, source attribution, and AMR	Genomic epidemiology/pathogen genomics
Broader across health domains; molecular public health applies an action-oriented framework specifically to infectious agents, hosts, and reservoirs [38,39].	More precisely targeted population prevention	Use of granular biological and population data to improve targeting and timing	Precision public health
End-to-end infectious-disease architecture; success is judged by actionability, equity, and measurable public-health impact.	Detection → interpretation → prediction → decision → intervention → evaluation	Integration of clinical, genomic, environmental, One Health, AMR, host-response, and computational signals	Molecular public health

**Table 2:** Core Bioinformatics Functions from Molecular Data to Public Health Intelligence.

Workflow Stage	Main Analytical Tasks	Typical Outputs	Why It Matters for Public Health Action	Key References
Preprocessing and quality control	Read quality assessment, trimming, decontamination, coverage review, and control checks	Cleaned reads, run metrics, pass/fail decisions	Ensures that reported molecular findings are reliable enough for downstream interpretation and decision-making	[21,22,27,44]
Assembly or mapping	Reference alignment or de novo assembly to reconstruct target genomes or genomic fragments	Consensus genomes, contigs, depth information, sequence completeness	Transforms raw sequencing output into analyzable molecular data	[13,14,22]
Annotation and classification	Lineage assignment, taxonomic profiling, mutation calling, virulence and AMR determinant detection	Lineages, clades, mutations, resistance genes, organism profiles	Provides biological meaning and supports comparison across time, place, and surveillance systems	[16,17,21,22]
Phylogenetics and cluster analysis	Relatedness assessment, cluster detection, transmission hypothesis generation, temporal analysis	Phylogenetic trees, genetic clusters, introduction or transmission hypotheses	Links molecular variation to epidemiological interpretation, outbreak investigation, and source attribution	[15,17,18,19,20,47,48,49,50]
Metadata integration and visualization	Linkage of sequence outputs with specimen date, place, host, exposure, and clinical context	Dashboards, risk summaries, standardized reports	Converts analytical outputs into actionable public health intelligence	[14,21,23,36,43,45]
Interpretive reporting	Uncertainty description, threshold-based flagging, and communication to incident-response teams	Alerts, action triggers, concise decision-ready summaries	Bridges laboratory findings with intervention such as IPC, targeted testing, vaccination, or policy adjustment	[21,23,36,43,45,46]

IPC, infection prevention and control; AMR, antimicrobial resistance.

**Table 3:** Major Molecular Technologies Used in Molecular Public Health.

Technology	Primary Molecular Signal	Key Strengths	Major Limitations	Representative Public Health Use	Key References
RT-qPCR/multiplex PCR	Defined pathogen-specific nucleic acid targets	Rapid, sensitive, scalable, and operationally mature; suited for routine diagnosis and surveillance	Requires predefined targets and provides limited information on genomic context	Case confirmation, respiratory or enteric surveillance, rapid trend monitoring	[3,10,11,12,55]
Digital PCR	Absolute target copy number	Improved precision at low concentration and relative tolerance to inhibitors	Lower throughput and still target-specific	Wastewater quantification, low-burden environmental surveillance, confirmatory measurements	[51]
Amplicon sequencing	Targeted genomic regions or near-complete viral genomes	Cost-effective lineage surveillance and variant tracking	Primer bias and limited value for unknown pathogens	SARS-CoV-2 and mpox genomic surveillance	[14,47,48,49,50]
Short-read whole-genome sequencing	Whole pathogen genomes	High base accuracy, mature analytical ecosystem, robust outbreak discrimination	May incompletely resolve plasmids or structural variants	Bacterial outbreak investigation, antimicrobial resistance surveillance, genomic epidemiology	[13,14,18,19,20,43]
Long-read sequencing/hybrid sequencing	Whole genomes plus structural context	Improved plasmid reconstruction, structural variant resolution, and portability	Higher analytical complexity and variable raw-read accuracy	AMR plasmid tracing, field sequencing, genome closure	[35,56]
Shotgun metagenomics	Unbiased nucleic acids from mixed samples	Pathogen-agnostic detection and ecological profiling in a single workflow	Cost, contamination sensitivity, and interpretive complexity	Unexplained syndromes, novel pathogen discovery, environmental and wastewater surveillance	[28,29,30,31,34,57,58,59]
CRISPR-based rapid molecular assays	Target-activated reporter signals	Rapid and potentially field-deployable	Sample processing, multiplexing, quantitative workflows, data capture, and standardization remain less mature than established PCR-based platforms	Decentralized outbreak testing and surge diagnostics	[52,53,54]

**Table 5:** Operational Metrics for Actionability in Molecular Public Health.

Illustrative Interpretation	What It Tests	Candidate Metric	Actionability Dimension
A technically unstable signal should not trigger intervention regardless of speed.	Whether the signal is reliable enough for interpretation	QC pass rate; sensitivity/specificity; reproducibility	Analytical validity
The same 48-h report may be timely for one use case and too late for another.	Whether information arrives while action can still alter outcomes	Sample-to-decision time; time /intervention-window ratio	Timeliness
Genomic similarity requires sampling, evolutionary-rate, and exposure context.	Whether molecular output supports the epidemiological inference	Cluster/source concordance; uncertainty; genotype–phenotype concordance	Interpretive reliability
Thresholds should reflect operational consequences, not AUROC alone.	Whether estimated risk is adequate for the intended trigger	Calibration; discrimination; sensitivity at the decision threshold	Predictive performance
A result that never reaches a decision owner is not operationally actionable.	Whether information changes public-health management	Reports changing/confirming action; time saved to action	Decision impact
Technical success without measurable added value may not justify continued resource use.	Whether the pathway improves outcomes beyond existing surveillance	Change in cases/transmission/shedding/resistance; incremental yield; resource use per useful decision	Outcome/incremental value

**Table 6:** Major Implementation Challenges and Practical Responses in Molecular Public Health.

Operational Challenge	Why It Limits Actionability	Potential Response or Implementation Strategy	Key References
Biased or incomplete sampling	Signals may not represent the underlying population, thereby distorting trend interpretation or cluster structure	Use transparent sampling frameworks, sentinel design, and complementary data streams such as wastewater surveillance	[36,43,45]
Insufficient metadata	Sequence data cannot be interpreted appropriately without time, place, host, and exposure context	Define minimum metadata standards and automate linkage wherever feasible	[21,23,36,43,45]
Long turnaround time	Action windows may close before results become available	Streamline workflows, automate reporting, and prioritize sample-to-report time as a core performance indicator	[19,20,43,46,47,56]
Bioinformatics capacity gaps	Data generation may outpace interpretation, delaying or weakening the public health response	Invest in workforce development, reproducible workflows, and regional or shared analytical infrastructure	[21,22,24,25,26,27,44,45,73,74]
Poor interoperability	Results are difficult to compare across institutions, sectors, or countries	Adopt shared analytical standards, controlled vocabularies, and interoperable reporting systems	[27,36,41,45,75,76]
Unclear action thresholds	Even strong molecular signals may not trigger intervention if decision rules are vague	Prospectively define trigger criteria linked to feasible interventions and revise them based on evaluation	[37,61,63]
Ethical, privacy, or governance concerns	Restrictions on data sharing may impede timely interpretation or cross-sector action	Use proportionate governance frameworks that protect privacy while preserving legitimate public health use	[36,41,76]
Unsustainable resource intensity	Capital and recurrent costs, bioinformatics burden, and maintenance needs can make a technically valid system nonviable.	Match technology to the decision use case; assess total cost of ownership and decision impact; regionalize sequencing/analysis; use targeted assays when sufficient.	[45,60,71,72,73,74]
Unequal capacity and data representativeness	Low-capacity regions contribute fewer samples, sequences, and metadata, biasing apparent lineage or resistance distributions.	Use sentinel designs, regional hubs, shared workflows, workforce investment, interoperable metadata, and equitable data-sharing/ benefit arrangements.	[36,41,72,73,74,75,76]
Failure of result-to- action translation	A valid molecular result may not change practice when reports are delayed, decision ownership is unclear, or response capacity is saturated.	Define decision owners and action windows prospectively; integrate reporting with response teams; measure decision impact and post-intervention outcomes	[19,20,43,45,63]

## Data Availability

No new data were created or analyzed in this study. Data sharing is not applicable to this article.

## References

[1] Falkow S. (1988). Molecular Koch’s postulates applied to microbial pathogenicity. Rev. Infect. Dis..

[2] Fredericks D.N., Relman D.A. (1996). Sequence-based identification of microbial pathogens: A reconsideration of Koch’s postulates. Clin. Microbiol. Rev..

[3] Gilbert G.L. (2002). Molecular diagnostics in infectious diseases and public health microbiology: Cottage industry to postgenomics. Trends Mol. Med..

[4] Drancourt M., Bollet C., Carlioz A., Martelin R., Gayral J.P., Raoult D. (2000). 16S ribosomal DNA sequence analysis of a large collection of environmental and clinical unidentifiable bacterial isolates. J. Clin. Microbiol..

[5] Seng P., Drancourt M., Gouriet F., La Scola B., Fournier P.E., Rolain J.M., Raoult D. (2009). Ongoing revolution in bacteriology: Routine identification of bacteria by matrix-assisted laser desorption ionization time-of-flight mass spectrometry. Clin. Infect. Dis..

[6] Poritz M.A., Blaschke A.J., Byington C.L., Allen L., Nilsson K., Jones D.E., Thatcher S.A., Robbins T., Lingenfelter B., Amiott E. (2011). FilmArray, an automated nested multiplex PCR system for multi-pathogen detection: Development and application to respiratory tract infection. PLoS ONE.

[7] Avery O.T., MacLeod C.M., McCarty M. (1944). Studies on the chemical nature of the substance inducing transformation of pneumococcal types. J. Exp. Med..

[8] Watson J.D., Crick F.H.C. (1953). Molecular structure of nucleic acids: A structure for deoxyribose nucleic acid. Nature.

[9] Sanger F., Nicklen S., Coulson A.R. (1977). DNA sequencing with chain-terminating inhibitors. Proc. Natl. Acad. Sci. USA.

[10] Saiki R.K., Scharf S., Faloona F., Mullis K.B., Horn G.T., Erlich H.A., Arnheim N. (1985). Enzymatic amplification of beta-globin genomic sequences and restriction site analysis for diagnosis of sickle cell anemia. Science.

[11] Mullis K.B., Faloona F.A. (1987). Specific synthesis of DNA in vitro via a polymerase-catalyzed chain reaction. Methods Enzymol..

[12] Higuchi R., Fockler C., Dollinger G., Watson R. (1993). Kinetic PCR analysis: Real-time monitoring of DNA amplification reactions. Biotechnology.

[13] Didelot X., Bowden R., Wilson D.J., Peto T.E.A., Crook D.W. (2012). Transforming clinical microbiology with bacterial genome sequencing. Nat. Rev. Genet..

[14] Armstrong G.L., MacCannell D.R., Taylor J., Carleton H.A., Neuhaus E.B., Bradbury R.S., Posey J.E., Gwinn M. (2019). Pathogen genomics in public health. N. Engl. J. Med..

[15] Hadfield J., Megill C., Bell S.M., Huddleston J., Potter B., Callender C., Sagulenko P., Bedford T., Neher R.A. (2018). Nextstrain: Real-time tracking of pathogen evolution. Bioinformatics.

[16] O’Toole Á., Scher E., Underwood A., Jackson B., Hill V., McCrone J.T., Colquhoun R., Ruis C., Abu-Dahab K., Taylor B. (2021). Assignment of epidemiological lineages in an emerging pandemic using the Pangolin tool. Virus Evol..

[17] Turakhia Y., Thornlow B., Hinrichs A.S., De Maio N., Gozashti L., Lanfear R., Haussler D., Corbett-Detig R. (2021). Ultrafast sample placement on existing trees (UShER) enables real-time phylogenetics for the SARS-CoV-2 pandemic. Nat. Genet..

[18] Vöhringer H.S., Sanderson T., Sinnott M., De Maio N., Nguyen T., Goater R., Schwach F., Harrison I., Hellewell J., Ariani C.V. (2021). Genomic reconstruction of the SARS-CoV-2 epidemic in England. Nature.

[19] Meredith L.W., Hamilton W.L., Warne B., Houldcroft C.J., Hosmillo M., Jahun A.S., Curran M.D., Parmar S., Caller L.G., Caddy S.L. (2020). Rapid implementation of SARS-CoV-2 sequencing to investigate cases of healthcare-associated COVID-19: A prospective genomic surveillance study. Lancet Infect. Dis..

[20] Stirrup O., Blackstone J., Mapp F., MacNeil A., Panca M., Holmes A., Machin N., Shin G.Y., Mahungu T., Saeed K. (2022). Effectiveness of rapid SARS-CoV-2 genome sequencing in supporting infection control for hospital-onset COVID-19 infection: Multicentre, prospective study. eLife.

[21] Cunningham-Oakes E., Trivett H. (2022). Applied bioinformatics and public health microbiology: Challenges, discoveries and innovations during a pandemic. Microb. Genom..

[22] Carriço J.A., Rossi M., Moran-Gilad J., Van Domselaar G., Ramirez M. (2018). A primer on microbial bioinformatics for nonbioinformaticians. Clin. Microbiol. Infect..

[23] Azarian T., Sherry N.L., Baker K., Holt K.E., Okeke I.N. (2022). Making microbial genomics work for clinical and public health microbiology. Microb. Genom..

[24] Köster J., Rahmann S. (2012). Snakemake—A scalable bioinformatics workflow engine. Bioinformatics.

[25] Di Tommaso P., Chatzou M., Floden E.W., Barja P.P., Palumbo E., Notredame C. (2017). Nextflow enables reproducible computational workflows. Nat. Biotechnol..

[26] Afgan E., Baker D., Batut B., van den Beek M., Bouvier D., Cech M., Chilton J., Clements D., Coraor N., Grüning B.A. (2018). The Galaxy platform for accessible, reproducible and collaborative biomedical analyses: 2018 update. Nucleic Acids Res..

[27] Ballard S.A., Sherry N.L., Howden B.P. (2023). Public health implementation of pathogen genomics: The role for accreditation and application of ISO standards. Microb. Genom..

[28] Chiu C.Y., Miller S.A. (2019). Clinical metagenomics. Nat. Rev. Genet..

[29] Gu W., Miller S., Chiu C.Y. (2019). Clinical metagenomic next-generation sequencing for pathogen detection. Annu. Rev. Pathol..

[30] Govender K.N., Street T.L., Sanderson N.D., Eyre D.W. (2021). Metagenomic sequencing as a pathogen-agnostic clinical diagnostic tool for infectious diseases: A systematic review and meta-analysis of diagnostic test accuracy studies. J. Clin. Microbiol..

[31] Gauthier N.P.G., Chan W., Locher K., Smailus D., Coope R., Charles M., Jassem A., Kopetzky J., Chorlton S.D., Manges A.R. (2024). Validation of an automated, end-to-end metagenomic sequencing assay for agnostic detection of respiratory viruses. J. Infect. Dis..

[32] Karthikeyan S., Levy J.I., De Hoff P., Humphrey G., Birmingham A., Jepsen K., Farmer S., Tubb H.M., Valles T., Tribelhorn C.E. (2022). Wastewater sequencing reveals early cryptic SARS-CoV-2 variant transmission. Nature.

[33] Smyth D.S., Trujillo M., Gregory D.A., Cheung K., Gao A., Graham M., Guan Y., Guldenpfennig C., Hoxie I., Kannoly S. (2022). Tracking cryptic SARS-CoV-2 lineages detected in NYC wastewater. Nat. Commun..

[34] Tisza M.J., Javornik Cregeen S., Avadhanula V., Zhang P., Ayvaz T., Feliz K., Hoffman K.L., Clark J.R., Terwilliger A., Ross M.C. (2023). Wastewater sequencing reveals community and variant dynamics of the collective human virome. Nat. Commun..

[35] Landman F., Jamin C., de Haan A., Witteveen S., Bos J., van der Heide H.G.J., Schouls L.M., Hendrickx A.P.A. (2024). Dutch CPE/MRSA Surveillance Study Group. Genomic surveillance of multidrug-resistant organisms based on long-read sequencing. Genome Med..

[36] World Health Organization (2022). Global Genomic Surveillance Strategy for Pathogens with Pandemic and Epidemic Potential, 2022–2032.

[37] World Health Organization (2024). Wastewater and Environmental Surveillance for One or More Pathogens: Guidance on Prioritization, Implementation and Integration.

[38] Khoury M.J., Bowen M.S., Clyne M., Dotson W.D., Gwinn M.L., Green R.F., Kolor K., Rodriguez J.L., Wulf A., Yu W. (2018). From public health genomics to precision public health: A 20-year journey. Genet. Med..

[39] Roberts M.C., Mensah G.A., Khoury M.J. (2024). Precision public health in the era of genomics and big data. Annu. Rev. Public Health.

[40] Djordjevic S.P., Jarocki V.M., Seemann T., Cummins M.L., Watt A.E., Drigo B., Wyrsch E.R., Reid C.J., Donner E., Howden B.P. (2024). Genomic surveillance for antimicrobial resistance: A One Health perspective. Nat. Rev. Genet..

[41] World Health Organization (2022). WHO Guiding Principles for Pathogen Genome Data Sharing.

[42] Islam M.S., Hasan M.M., Wang X., Germack H.D., Noor-E-Alam M. (2018). A systematic review on healthcare analytics: Application and theoretical perspective of data mining. Healthcare.

[43] Struelens M.J., Ludden C., Werner G., Sintchenko V., Jokelainen P., Ip M. (2024). Real-time genomic surveillance for enhanced control of infectious diseases and antimicrobial resistance. Front. Sci..

[44] Ewels P., Magnusson M., Lundin S., Käller M. (2016). MultiQC: Summarize analysis results for multiple tools and samples in a single report. Bioinformatics.

[45] Ferdinand A.S., Kelaher M., Lane C.R., Gonçalves da Silva A., Sherry N.L., Ballard S.A., Andersson P., Hoang T., Denholm J.T., Easton M. (2021). An implementation science approach to evaluating pathogen whole genome sequencing in public health. Genome Med..

[46] Sundermann A.J., Rosa R., Harris P.N.A., Snitkin E., Javaid W., Moore N.M., Hayden M.K., Allen K., Rodino K., Peacock S.J. (2025). Pathogen genomics in healthcare: Overcoming barriers to proactive surveillance. Antimicrob. Agents Chemother..

[47] Oude Munnink B.B., Nieuwenhuijse D.F., Stein M., O’Toole Á., Haverkate M., Mollers M., Kamga S.K., Schapendonk C., Pronk M., Lexmond P. (2020). Rapid SARS-CoV-2 whole-genome sequencing and analysis for informed public health decision-making in the Netherlands. Nat. Med..

[48] du Plessis L., McCrone J.T., Zarebski A.E., Hill V., Ruis C., Gutierrez B., Raghwani J., Ashworth J., Colquhoun R., Connor T.R. (2021). Establishment and lineage dynamics of the SARS-CoV-2 epidemic in the UK. Science.

[49] Volz E., Mishra S., Chand M., Barrett J.C., Johnson R., Geidelberg L., Hinsley W.R., Laydon D.J., Dabrera G., O’Toole Á. (2021). Assessing transmissibility of SARS-CoV-2 lineage B.1.1.7 in England. Nature.

[50] Isidro J., Borges V., Pinto M., Sobral D., Santos J.D., Nunes A., Mixão V., Ferreira R., Santos D., Duarte S. (2022). Phylogenomic characterization and signs of microevolution in the 2022 multi-country outbreak of monkeypox virus. Nat. Med..

[51] Sancha Dominguez L., Cotos Suárez A., Sánchez Ledesma M., Muñoz Bellido J.L. (2024). Present and future applications of digital PCR in infectious diseases diagnosis. Diagnostics.

[52] Fozouni P., Son S., Díaz de León Derby M., Knott G.J., Gray C.N., D’Ambrosio M.V., Zhao C., Switz N.A., Kumar G.R., Stephens S.I. (2021). Amplification-free detection of SARS-CoV-2 with CRISPR-Cas13a and mobile phone microscopy. Cell.

[53] Ghouneimy A., Mahas A., Marsic T., Aman R., Mahfouz M. (2023). CRISPR-based diagnostics: Challenges and potential solutions toward point-of-care applications. ACS Synth. Biol..

[54] Shen Y., Hu K., Yuan M., Duan G., Guo Y., Chen S. (2023). Progress and bioapplication of CRISPR-based one-step, quantitative and multiplexed infectious disease diagnostics. J. Appl. Microbiol..

[55] Peeling R.W., Heymann D.L., Teo Y.Y., Garcia P.J. (2022). Diagnostics for COVID-19: Moving from pandemic response to control. Lancet.

[56] Quick J., Loman N.J., Duraffour S., Simpson J.T., Severi E., Cowley L., Akoi Bore J., Koundouno R., Dudas G., Mikhail A. (2016). Real-time, portable genome sequencing for Ebola surveillance. Nature.

[57] Downie D.L., Rao P., David-Ferdon C., Courtney S., Lee J.S., Kugley S., MacDonald P.D.M., Barnes K., Fisher S., Andreadis J.L. (2024). Literature review of pathogen agnostic molecular testing of clinical specimens from difficult-to-diagnose patients: Implications for public health. Health Secur..

[58] Morton L., Creppage K., Rahman N., Early J., Hartman L., Hydrick A., Kasper M. (2024). Challenges and opportunities in pathogen agnostic sequencing for public health surveillance: Lessons learned from the Global Emerging Infections Surveillance Program. Health Secur..

[59] Minor N.R., Ramuta M.D., Stauss M.R., Harwood O.E., Brakefield S.F., Alberts A., Vuyk W.C., Bobholz M.J., Rosinski J.R., Wolf S. (2023). Metagenomic sequencing detects human respiratory and enteric viruses in air samples collected from congregate settings. Sci. Rep..

[60] Gador-Whyte A.P., Sherry N.L., Brischetto A., Andersson P., Bond K.A., van Hal S.J., Harris P.N.A., Howden B.P., Australian Pathogen Genomics Program partners (2026). Implementation of pathogen genomics in clinical microbiology laboratories. Clin. Microbiol. Rev..

[61] National Academies of Sciences, Engineering, and Medicine (2023). Wastewater-Based Disease Surveillance for Public Health Action.

[62] Boogaerts T., Van Wichelen N., Quireyns M., Burgard D., Bijlsma L., Delputte P., Gys C., Covaci A., van Nuijs A.L.N. (2024). Current state and future perspectives on de facto population markers for normalization in wastewater-based epidemiology: A systematic literature review. Sci. Total Environ..

[63] de Jong M., de Korne-Elenbaas J., Fanoy E., Medema G., de Graaf M., Prins M., Schim van der Loeff M.F., Daams J., de Roda Husman A.M., Heijne J.C.M. (2026). Public health actions in response to pathogen detection in wastewater and the environment: A scoping review. Front. Public Health.

[64] Carleton H.A., Gerner-Smidt P. (2016). Whole-genome sequencing is taking over foodborne disease surveillance. Microbe.

[65] World Health Organization (2025). Global Antibiotic Resistance Surveillance Report 2025.

[66] Kim J.I., Maguire F., Tsang K.K., Gouliouris T., Peacock S.J., McAllister T.A., McArthur A.G., Beiko R.G. (2022). Machine learning for antimicrobial resistance prediction: Current practice, limitations, and clinical perspective. Clin. Microbiol. Rev..

[67] Overmyer K.A., Shishkova E., Miller I.J., Balnis J., Bernstein M.N., Peters-Clarke T.M., Meyer J.G., Quan Q., Muehlbauer L.K., Trujillo E.A. (2021). Large-scale multi-omic analysis of COVID-19 severity. Cell Syst..

[68] Su Y., Chen D., Yuan D., Lausted C., Choi J., Dai C.L., Voillet V., Duvvuri V.R., Scherler K., Troisch P. (2020). Multi-omics resolves a sharp disease-state shift between mild and moderate COVID-19. Cell.

[69] Villanueva-Miranda I., Xiao G., Xie Y. (2025). Artificial intelligence in early warning systems for infectious disease surveillance: A systematic review. Front. Public Health.

[70] Li J.-H., Tseng Y.-J., Chen S.-H., Chen K.-F. (2026). Artificial intelligence in infection surveillance: Data integration, applications and future directions. Biomed. J..

[71] Tran M., Smurthwaite K.S., Nghiem S., Cribb D.M., Zahedi A., Ferdinand A.D., Andersson P., Kirk M.D., Glass K., Lancsar E. (2023). Economic evaluations of whole-genome sequencing for pathogen identification in public health surveillance and health-care-associated infections: A systematic review. Lancet Microbe.

[72] Yek C., Pacheco A.R., Vanaerschot M., Bohl J.A., Fahsbender E., Aranda-Díaz A., Lay S., Chea S., Oum M.H., Lon C. (2022). Metagenomic pathogen sequencing in resource-scarce settings: Lessons learned and the road ahead. Front. Epidemiol..

[73] Mboowa G., Kakooza F., Egesa M., Tukwasibwe S., Kanyerezi S., Sserwadda I., Kidenya B.R., Kabahita J.M., Namaganda M.M., Nsubuga M. (2024). The rise of pathogen genomics in Africa. F1000Research.

[74] Onywera H., Ondoa P., Nfii F., Ogwell A., Kebede Y., Christoffels A., Tessema S.K. (2024). Boosting pathogen genomics and bioinformatics workforce in Africa. Lancet Infect. Dis..

[75] Khare S., Gurry C., Freitas L., Schultz M.B., Bach G., Diallo A., Akite N., Ho J., Lee R.T.C., Yeo W. (2021). GISAID’s role in pandemic response. China CDC Wkly..

[76] Halabi S., Wilder R.C., Gostin L.O., Hurtado M.L. (2023). Sharing pathogen genomic sequence data—Toward effective pandemic prevention, preparedness, and response. N. Engl. J. Med..

